# Comparison of widefield swept-source optical coherence tomography angiography with ultra-widefield fluorescein angiography for the evaluation of lesions in retinal vein occlusion

**DOI:** 10.1186/s12886-022-02642-1

**Published:** 2022-11-07

**Authors:** Li Siying, Zeng Qiaozhu, Han Xinyao, Zhang Linqi, Zhao Mingwei, Qu Jinfeng

**Affiliations:** Department of Ophthalmology, Eye Diseases and Optometry Institute; Beijing Key Laboratory of Diagnosis and Therapy of Retinal and Choroid Diseases, College of Optometry, Peking University People’s Hospital, Peking University Health Science, No. 11 Xizhimen South Street, Xicheng District, Beijing, China

**Keywords:** Widefield SS-OCTA, Ultra-widefield FA, Retinal vein occlusion lesions, Diagnosis and monitoring

## Abstract

**Background:**

To compare widefield swept-source optical coherence tomography angiography (SS-OCTA) with ultra-widefield fundus fluorescein angiography (UWF-FA) for detecting retinal vein occlusion (RVO) lesions.

**Methods:**

Thirty-four eyes of 32 patients with treatment-naïve RVO were enrolled at Peking University People’s Hospital from September 2021 to March 2022. Patients were imaged with a UWF-FA (200°) and a widefield SS-OCTA using 24 × 20 mm scan single capture. Quantitative assessments of RVO lesions such as foveal avascular zone (FAZ) area and perimeter, non-perfusion areas (NPA), number of microaneurysms (MAs), capillary changes and collateral vessels were performed.

**Results:**

The measurement of FAZ area and perimeter were comparable between SS-OCTA and UWF-FA (0.373 (range, 0.277–0.48) mm^2^ vs. 0.370 (range, 0.277–0.48) mm^2^, *P* = 0.818 and 2.480 (range, 2.011–2.998) vs. 2.330 (range, 2.027–2.807) mm, *P* = 0.536, respectively). Intraclass correlation coefficients (ICCs) of FAZ area and perimeter between SS-OCTA and UWF-FA was high (0.999, [0.997–0.999] and 0.996 [0.991–0.996], respectively), suggesting good agreement. The mean NPA area was larger on SS-OCTA than that on UWF-FA (89.977 ± 78.805 mm^2^vs. 87.944 ± 77.444 mm^2^, *P* = 0.037). The ICC of NPA area was also high (0.999, [0.999–1.000]). The median of total MA count was less on SS-OCTA than on UWF-FA (7 (range, 0–19) vs.12 (range, 0–23), *P* < 0.001). Agreement in detecting MAs between SS-OCTA and UWF-FA was found to be good (ICC = 0.920, [0.555–0.974]).The total capillary changes and collateral vessels count were less on UWF-FA than SS-OCTA (11 ± 9 vs 6 ± 7, *P* < 0.001 and 4 (range, 0–6) vs 0 (range, 0–0), *P* < 0.001, respectively). Agreement in detecting capillary changes and collateral vessels between OCTA and UWF-FA was found to be fair (ICC = 0.733, [0.081–0.905] and 0.564, [0.039–0.805], respectively).

**Conclusion:**

Compared with UWF-FA, widefield SS-OCTA was found comparable or even superior in detecting FAZ, NPA, capillary changes and collateral vessels except MAs in RVO. Widefield SS-OCTA may offer a more efficient alternative to FA for diagnosis and monitoring RVO.

## Background

Retinal vein occlusion (RVO) is the second most common retinal vascular disorder next to diabetic retinopathy [1]. Its common complications include macula edema, retinal ischemia, neovascularization, etc., which are significant causes of severe vision loss [2]. Therefore, reliable identification and adequate measurement of the abnormalities promotes early intervention and helps to avoid visual impairment.

Fluorescein angiography (FA) has been critically significant in determining whether RVO is ischemic and deciding the necessity of laser photocoagulation treatment [3]. However, it is an invasive, time-consuming and relatively expensive procedure that can cause nausea, vomiting, and even anaphylaxis. Moreover, it cannot be performed neither in pregnant women, patients with allergy to fluorescein, renal failure, severe asthma or cardiac disease nor on the same day [4].

Optical coherence tomography angiography (OCTA) is a non-invasive, time-saving, high-resolution method which can clearly depict and quantitatively analyze the microvascular abnormalities of different layers of retina and choroid [5]. Previous OCTA platforms were mainly focused on the macular area which restricts their application in retinal vascular disease since many of them have lesions beyond macular area. Recently, a novel widefield SS-OCTA device BM400K is available from TowardPi Medical Technology in China. With the combination of long wavelength(1060 nm)full range swept source and 400 kHz A-scan rate, the device has capability to acquire as deep as 6 mm scan depth and a wide scan area as 24mmx20mm (81° × 68°) in a single capture, which may potentially change the paradigm of FA-based diagnosis, grading and follow-up of RVO.

In this observational cross-sectional study, we compared widefield SS-OCTA with ultra-widefield angiography (UWF-FA) to detect RVO lesions such as foveal avascular zone (FAZ) area and perimeter, non-perfusion areas (NPA), number of microaneurysms (MAs), capillary changes, collateral vessels and retinal neovascularization.

## Methods

### Patients

This cross-sectional investigation enrolled consecutively patients with central retinal vein occlusion (CRVO) or branch retinal vein occlusion (BRVO) who visited the outpatient clinic of Peking University People’s Hospital between September 2021 and March 2022. The inclusion criteria were diagnosis of RVO (central or branch of the retinal vein) based on clinical evaluation and fundus retinal exam performed by at least two retina specialists. Exclusion criteria were as follows: 1) The eyes with any history of ocular injury, ocular surgery, retinal laser photocoagulation, and other retinal, optic nerve, and choroidal diseases that may confound the results; 2) Participants with severe media opacities and refractive error (more than 6 diopters of sphere or more than 3 diopters of cylinder) that may interfere images acquisition were also excluded; 3) Besides, SS-OCTA images with signal strength index < 7 or severe artifacts preventing accurate analysis were excluded.

This study was approved by the institutional review board of Peking University People’s Hospital, and informed consent was obtained from all subjects. All the procedures adhered to the tenets of the Declaration of Helsinki.

### Study protocol

Data on baseline demographics and systematic conditions like course of disease and arterial hypertension were recorded. All enrolled patients underwent a full ophthalmic examination, including automatic refractometry, measurement of best corrected distance visual acuity (BCVA), intraocular pressure (IOP), slit lamp examination, indirect ophthalmoscopy, color fundus photography, UWF-FFA using Optos 200Tx (Optos plc, Dunfermline, United Kingdom) and a single capture of 24 × 20 mm widefield SS-OCTA scan using BM400K (BMizar, TowardPi Medical Technology Co., Ltd, Beijing, China) on the same day. We compared the en-face OCTA images of superficial capillary plexus (SCP) slab, which is between the inner limiting membrane and 9 µm above the inner plexiform layer with venous-phase images of UWF-FFA.

### Image processing and analysis

Two masked ophthalmologists (Li Siying and Zeng Qiaozhu) independently evaluated CFP, FA and SS-OCTA images. A third well-trained ophthalmologist (Qu Jinfeng) adjudicated cases with discrepancies. OCTA images were segmented automatically using the built-in software and the segmentation errors were corrected manually if necessary.

**Fig. 1:**
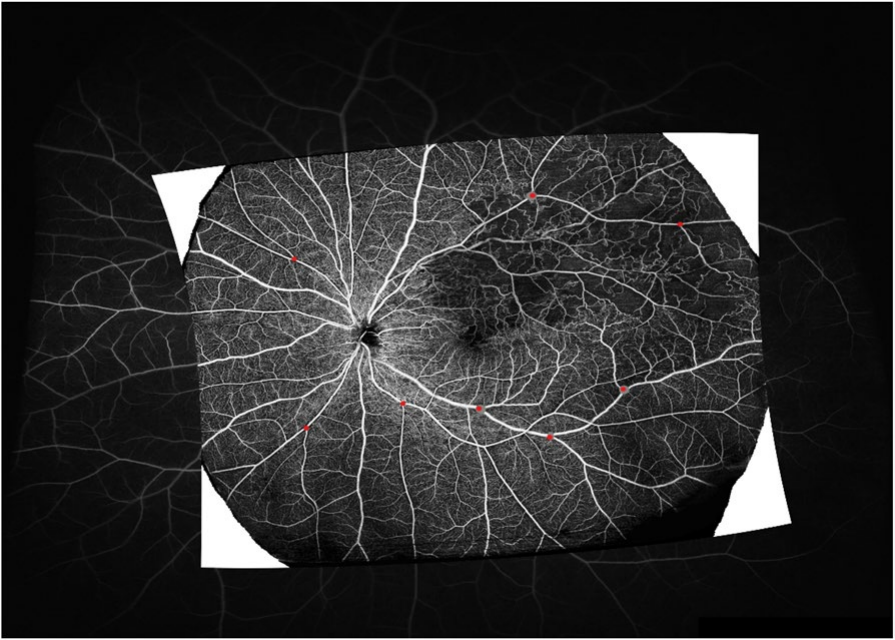
A representative example of registered images with multiple red corresponding points

En face SS-OCTA images of SCP slab and representative UWF-FFA images were exported in TIF formats and registrated -using Image J software (version 1.4.3.67, 2016) and the Landmark Correspondences plug-in (http://imagej.net/Landmark_Correspondences). Specifically, we manually chose several landmarks on the two images that definitely corresponded with each other (e.g., vessel bifurcation or arteriovenous crossings), and performed the non-linear image transformation of the OCTA image by matching the corresponding landmarks on UWF-FA image as the reference described [6]. A representative example of registration is presented in Fig. 1. The registered SS-OCTA images and the corresponding same area of UWF-FA images were exported and then imported into Image J for the following quantitative analysis and comparison.

**Fig. 2:**
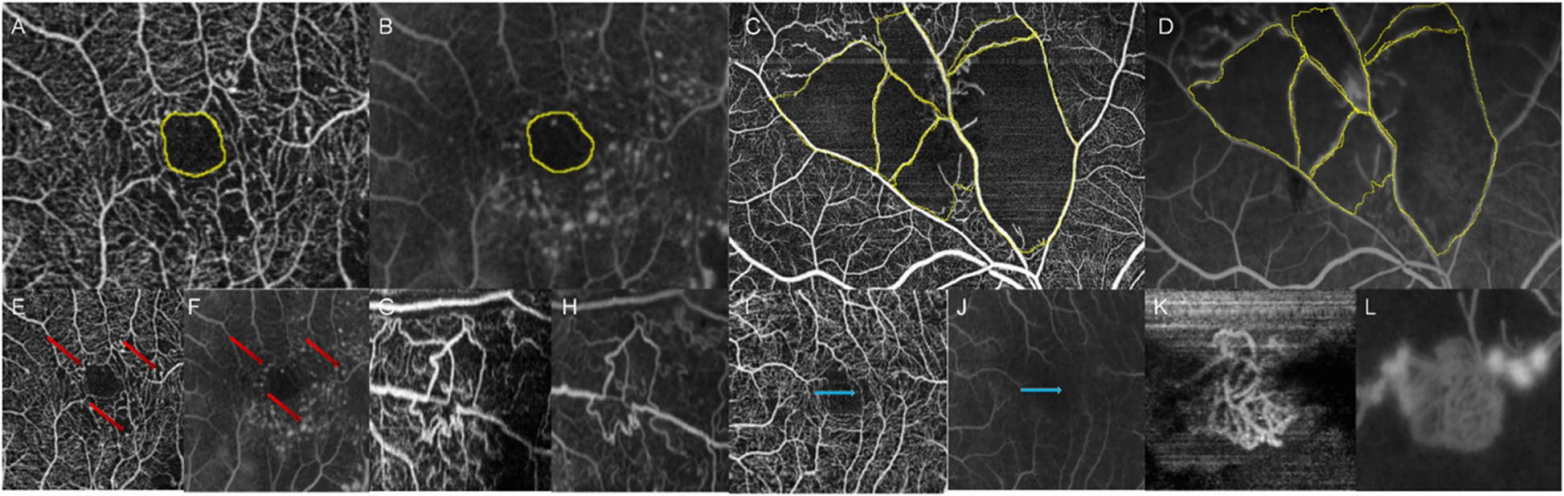
Comparison of representative lesions on SS-OCTA and corresponding UWF-FA images of RVO patients. Foveal avascular zone (FAZ) and non-perfusion area (NPA) were outlined on OCTA (**A** and **C**) and UWF-FA images (**B** and **D**). **E** and **F** showed cluster of microaneurysms (MAs) around central fovea (red arrows) on OCTA and FA. Capillary changes were displayed in **G** and **H**. Collateral vessels (blue arrows) were shown in **I** and **J**. A clump of neo-vascularization was shown on **K** and **L**

The FAZ area, FAZ perimeter and NPAs on UWF-FA was traced manually and measured by using built-in OptosAdvance® software of Optos 200Tx*.*The FAZ area and FAZ perimeter on SS-OCTA were measured by manually outlining the FAZ margin using Image J (Fig. 2A-B). NPAs which appeared as an area of the fundus devoid of retinal arterioles, venules, and capillaries, with a pruned appearance of adjacent vessels on FFA, and as the absence of capillary bed between a terminal arteriole and a proximal venule or larger vessels on SS-OCTA were manually traced and measured in each image by using Image J (Fig. 2C-D) [7]. Considering the effect of axial length-related magnification, we used formula *t* = *p. q. s* to make an axial length correction as described by previous publication for SS-OCTA measurement [8]. In this formula, *t* is the actual fundus dimension, *s* is the measurement on OCTA, *p* is a constant in a telecentric system which can be calculated as 3.382, and *q* is the magnification factor related to the eye which can be calculated as *q* = 0.01306 (*x-*1.82), where *x* is the axial length [9].The number of microaneurysms (MAs),capillary changes, collateral vessels and retinal neovascularizations (NVs) in each eye were counted in both modalities (Fig. 2E-L).

### Statistical analysis

All statistical analyses were performed with Stata/SE 15.0 (V.15.0; Stata, College Station, TX, USA). Normally distributed continuous variables were presented as mean ± SD. Nonnormally distributed continuous variables were presented as median and range. Student’s t test (t test) was performed to compare normally distributed quantitative variables, while the nonparametric Wilcoxon signed rank test was used for abnormally distributed quantitative variables. The intraclass correlation coefficient (ICC) and its 95% confidence interval (CI) were used to assess the agreement between SS-OCTA and UWF-FA pairwise for continuous variables. The ICC (range, 0–1) of 0.75 to 1 was accepted as good agreement, 0.5 to 0.75 as fair agreement, and below 0.5 as poor agreement [10]. The agreement between the two imaging modalities was also assessed by Bland–Altman plots. A *P* value of 0.05 was considered to be statistically significant.

## Results

### Demographics

Thirty-four eyes of 32 patients were enrolled in this study, including 17 (53%) males and 15 (47%) females. All patients were Han nationality from Chinese mainland. Demographics and clinical characteristics are provided in Table 1. There were 12 (35%) eyes with CRVO and 22 (65%) eyes with BRVO. Mean age was 56.3 ± 14.4 years. Twenty-five (78%) patients had hypertension. The mean duration of the symptoms of RVO was 2.1 ± 1.4 months. The mean BCVA in logarithm of minimum angle of resolution (log MAR) was 0.39 ± 0.22, and the mean IOP was 17.3 ± 2.9 mmHg.

**Table 1:** Demographic and clinical characteristics of included patients

Parameters	Value
Demographic characteristics	
Age, years, mean (±SD)	56.3 (14.4)
Sex (male: female)	17:15
Disease duration, months, mean (±SD)	2.1 (1.4)
Hypertension, n (%)	25 (78.1)
Ocular profile	
Eyes involved, n (%)	
Unilateral	30 (93.75)
Bilateral	2 (6.25)

### Quantitative assessment of lesions

#### Foveal avascular zone

**Fig. 3:**
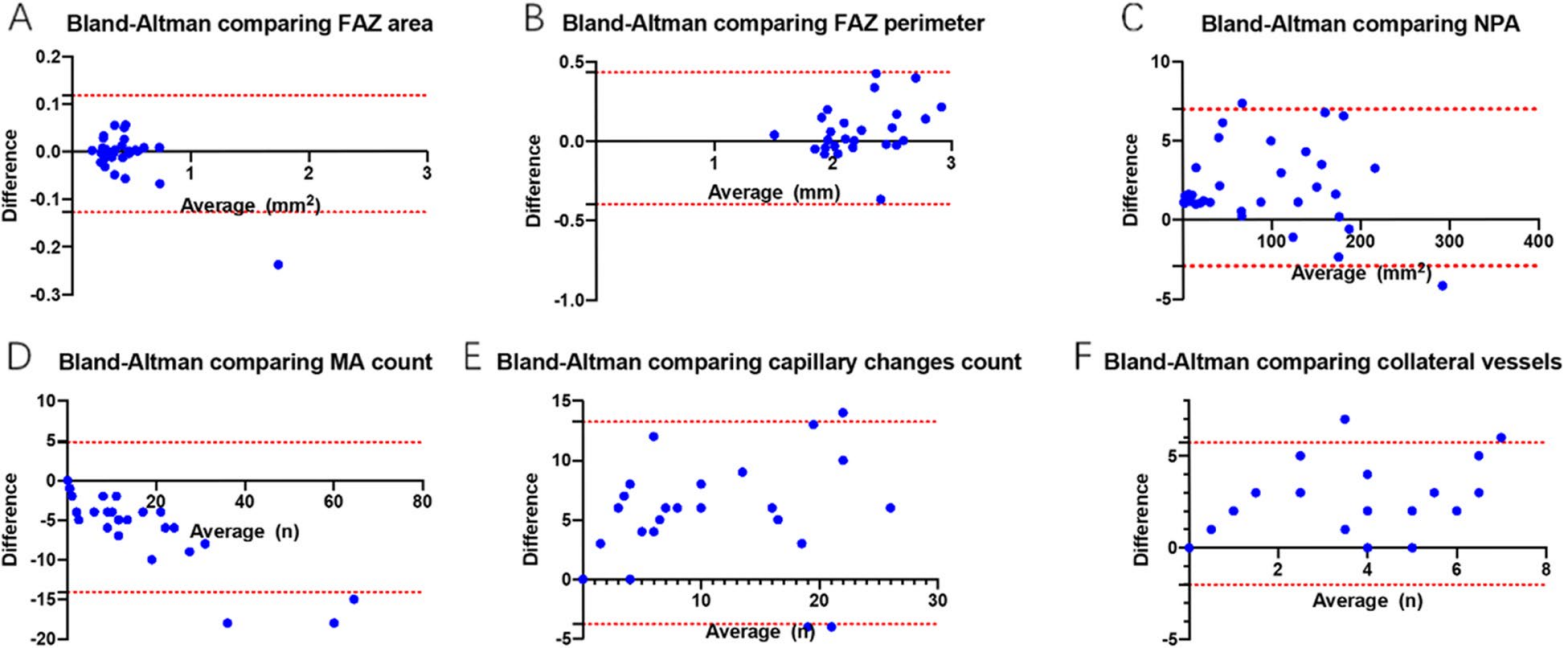
Bland–Altman analysis of RVO lesions. FAZ area (**A**), FAZ perimeter (**B**), NPA (**C**), counts of MA (**D**), counts of capillary changes (**E**) and counts of collateral vessels (**F**) in SS-OCTA and FA

The median FAZ area was 0.373 (range, 0.277–0.48) mm^2^ on SS-OCTA and 0.370 (range, 0.277–0.48) mm^2^ on UWF-FA, while the median FAZ perimeter was 2.480 (range, 2.011–2.998) and 2.330 (range, 2.027–2.807) mm on SS-OCTA and UWF-FA images respectively. No significant difference was found in FAZ area and perimeter between SS-OCTA and UWF-FA images (*P* = 0.818 and 0.536, respectively) (Table 2). ICCs of FAZ area and perimeter between SS-OCTA and UWF-FA was high (0.999, [0.997–0.999] and 0.996 [0.991–0.996], respectively), suggesting good agreement between the two imaging modalities (Table 3). The good consistency was also demonstrated using Bland–Altman plots (Fig. 3A-B).

**Table 2:** Detection of RVO lesions on SS-OCTA versus UWF-FA

Lesions	SS-OCTA	UWF-FA	*P*-value
FAZ area, mm^2^, median (IQR)	0.373 (0.277–0.48)	0.370 (0.277, 0.48)	0.818
FAZ perimeter, mm, median (IQR)	2.480 (2.011–2.998)	2.330 (2.027, 2.807)	0.536
NPA, mm^2^, mean (± SD)	89.977 (78.805)	87.944 (77.444)	0.037*
MA, n, median (IQR)	7 (0, 19)	12 (0, 23)	< 0.001**
Capillary changes, n, mean (± SD)	11 (9)	6 (7)	< 0.001**
Collateral vessel, n, median (IQR)	4 (0, 6)	0 (0, 0)	< 0.001**

*RVO* retinal venous occlusion, *SS-OCTA* swept-source optical coherence tomography, *UWF-FA* ultra-widefield fluorescein angiography, *FAZ* foveal avascular zone, *NPA* nonperfusion area, *MA* microaneurysm, *IQR* interquartile range, *SD* standard deviation

**Table 3:** The ICCs of RVO lesions between SS-OCTA and UWF-FA

Lesions	ICC	95%CI	*P*-value
FAZ area	0.999	0.997–0.999	< 0.001
FAZ perimeter	0.996	0.991–0.996	< 0.001
NPA	0.999	0.999–1.000	< 0.001
MA count	0.920	0.555–0.974	< 0.001
capillary changes count	0.733	0.081–0.905	< 0.001
collateral vessel count	0.564	0.039–0.805	< 0.001

*RVO* retinal venous occlusion, *SS-OCTA* swept-source optical coherence tomography, *UWF-FA* ultra-widefield fluorescein angiography, *FAZ* foveal avascular zone, *NPA* nonperfusion area, *MA* microaneurysm, *ICC* intraclass correlation coefficient, *CI* confidence interval

### Nonperfusion area

The mean NPA area was larger on SS-OCTA than that on UWF-FA (89.977 ± 78.805 vs. 87.944 ± 77.444 mm^2^, *P* = 0.037) as presented in Table 2. The ICC of NPA area was also high (0.999, [0.999–1.000]) (Table 3). A Bland–Altman plot comparing the measurement of NPA area on SS-OCTA and UWF-FA showed good consistency (Fig. 3C).

### Counts of MA, capillary changes, collateral vessels and NVs

The median of MA count in each eye was 7 (range, 0–19) on SS-OCTA compared with 12 (range, 0–23) on UWF-FA. UWF-FA showed better performance in detecting MAs (*P* < 0.001) (Table 2). Agreement in detecting MAs between widefield SS-OCTA and UWF-FA was good (ICC = 0.920, [0.555–0.974]) (Table 3). Three eyes showed the value beyond the 95% confidence interval of mean difference of MAs count on SS-OCTA and UWF-FA in Bland–Altman plot (Fig. 3D).

The mean count of capillary changes in each eye was 11 ± 9 on SS-OCTA compared to 6 ± 7 on UWF-FA. SS-OCTA showed better performance in detecting capillary changes (*P* < 0.001) (Table 2). Agreement in detecting capillary changes between widefield SS-OCTA and UWF-FA was found to be fair (ICC = 0.733, [0.081–0.905]) (Table 3). Three eyes showed value beyond the 95% confidence interval of mean difference of capillary changes count on SS-OCTA and UWF-FA in Bland–Altman plot (Fig. 3E).

The median count of collateral vessels in each eye was 4 (range, 0–6) on SS-OCTA compared with 0 on UWF-FA. SS-OCTA performed better in detecting collateral vessels (*P* < 0.001) (Table 2). Agreement in detecting collateral vessels between widefield SS-OCTA and UWF-FA was found to be fair (ICC = 0.564, [0.039–0.805]) (Table 3). Two eyes showed value beyond the 95% confidence interval of mean difference of collateral vessels count on SS-OCTA and UWF-FA in Bland–Altman plot (Fig. 3F).

Both SS-OCTA and UWF-FA detected 6 retinal NVs in 4 eyes. Agreement in detecting NV between OCTA and FA was found to be excellent (ICC = 1).

## Discussion

In this study, we compared the differences in detecting microvascular lesions, including FAZ, NPAs, MAs, capillary changes, collateral vessels and NVs, between widefield SS-OCTA (24 × 20 mm) and UWF-FA in treatment-naïve RVO eyes and found that compared with UWF FA, widefield SS-OCTA was comparable or even superior in detecting FAZ, NPA, capillary changes and collateral vessels except MAs in RVO.

The FAZ is the macular capillary-free zone surrounded by interconnected capillary vessels. Previous studies have proposed a mean physiological FAZ area of 200 to 400mm^2^in healthy subjects [11]. As is well known that FAZ enlargement indicate macular ischemia and poor visual acuity. Coscas et al. found that when the arcade was intact on OCTA, FA never showed peripheral ischemia [12]. Therefore, measurements of the FAZ could help to objectively evaluate degree of disease and prognosis. Previous studies have come up with results that there was good consistency between FA and OCTA for FAZ grading, although their study did not quantitate this observation [13, 14]. Differed from them, we quantified the areas and perimeters of FAZ in order to get more precise measurement data and reliable statistical results. Similar methods have been reported in other studies [15, 16]. After quantification, we reached similar conclusion that the mean sizes and perimeters of the FAZ in the same eye were equal between OCTA and FA measurements. Therefore, it was assumed that widefield SS OCTA may be an alternative to FA to evaluate FAZ in RVO patients.

Retinal vein occlusion is a precipitating event that leads to baseline ischemia and release of the vascular endothelial growth factor (VEGF), which then contributes to progression of NPA and thus worsening of ischemia [17].Nonperfused areas (NPAs) form in relation with retinal artery or vein occlusion, which is a significant sign for ischemia. We generally consider that NPAs > 5 discs areas in branch retinal vein occlusion (BRVO) and NPAs > 10 discs areas in central retinal vein occlusion (CRVO) as ischemia types, which means high risk of neovascularization progression and severe vision deterioration [18]. Several studies have confirmed that large or progressive NPAs were closely related to NV. In other words, NPA may be seen as a biomarker for the assessment of disease progression or regression [19, 20]. Therefore, identification of retinal NPA can not only influence the management and treatment of RVO but also indicate the prognosis after treatment. Previous literatures found that OCTA has similar or better sensitivity to detect NPA compared with FA [14, 21], but the quantification of NPA was not described in their studies. In our study, areas of NPAs were manually sketched and quantified on Image J. Our results suggested that the NPA on SS-OCTA images were slightly larger than that on UWF-FA, which was consistent with previous investigations. This difference can be explained by no interference of fluorescence leakage on OCTA due to a high resolution of vascular network in macular images on OCTA. Moreover, blockages of fluorescence due to retinal hemorrhage on FA can be avoided on OCTA images due to its long wavelength. But it should be cautious that the void of vessels on OCTA may be caused not only by the nonperfusion but also very slow or turbulence of blood flow which was under device’s detection threshold.

Differed from diabetic retinopathy (DR), MAs in RVO usually occurs in the chronic stage which usually form at the border of NPAs or around collateral vessels [22, 23]. A study showed that the microaneurysms appeared 6 months after disease onset [24]. In our study, MA count was significantly less on SS-OCTA than on UWF-FA. It mainly resulted from the fact that in FA the dye remains in the abnormally dilated blood vessel, leading to the brightly and exaggerate hyperfluorescent appearances [25]. However, high-speed SS-OCTA could limit the identification of low-flow lesions such as MAs and can’t show dye leakage.

In eyes with RVO, venous obstruction resulted in turbulent blood flow, elevated venous pressure and overload of drainage capacity that may make small vessels and capillaries dilated, tortuous and frizzy. Capillary changes present as dilated vessels or capillary telangiectasia usually arose between the affected and healthy venules, which were more common in eyes with major RVO or the ischemic type than those with macular BRVO or the nonischemic type [26, 27]. In accordance with previous studies, we found more capillary changes in SS-OCTA than UWF-FA. Widefield SS-OCTA can visualize microvascular abnormalities better than UWF-FA in RVO eyes due to its high image resolution and low obscuration [14, 21, 28]. The collateral vessels were presented as a long vessel traversing the area with blocked perfusion, or as a bunch of tortuous vessels in the vicinity of the area with blocked perfusion [29]. Its formation was speculated to assist in draining the obstructed venous flow into the non-obstructed area, tending to decrease the retinal venous pressure in the occluded segment. Suzuki et al. found that macular edema in eyes with collateral vessels were more quickly and frequently resolved than that in eyes without collateral vessels. Moreover, the mean CRT reduction at 6 months after treatments was significantly greater than in eyes without collaterals [26]. In our study, more collateral vessels were found in SS-OCTA than UWF-FA, which was similar to previous study [21, 26].This phenomenon was also due to high resolution on OCTA and no interference of fluorescence blockage or leakage.

Kadomoto and associates also reported the comparison between widefield OCTA and Optos FA measuring NPA and number of retinal neovascularization in BRVO cases and they found that for patients with accompanying neovascularization, the retinal NPAs were larger than those without neovascularization [19]. FA is a useful and important tool to confirm NV by dye diffusion, but the structure of NV is limited to two dimensions. In this study, a scan size of 24 × 20 mm was obtained which was the largest scan range among all the commercially available SS-OCTA devices. It provides us not only a 3-dimensional non-invasive view of microvascular structure but also 2.4 times enlargement of the single-center OCTA FOV. This advantage may potentially change the paradigm of diagnosis and follow-up of RVO based on FA.

There are several limitations in our study. First, we included only a small number of eyes, and larger prospective series are needed to further confirm these results. Second, the manual segmentation and annotation methods could cause measurement bias. Third, as it was only a cross-sectional study with imaging captured at a single time point, a follow-up study with longitudinal data would be even more helpful for determining the role of SS-OCTA for RVO. Fourth, this was a study carried out in Chinese patients only, whether similar results can be found in other ethnics still need further investigation.

## Conclusions

Compared with UWF-FA, widefield SS-OCTA (24 × 20 mm) was found comparable or even superior in detecting FAZ, NPA, capillary changes and collateral vessels except MAs in RVO. Since OCTA is a noninvasive, time-saving, easy-to-use and quantitative imaging technique, widefield SS-OCTA may offer a more efficient alternative to FA for diagnosis and monitoring of RVO.

## Data Availability

The data are available with the corresponding author.

## Abbreviations

SS-OCTA: Swept-source optical coherence tomography angiography
UWF FA: Ultra-widefield fundus fluorescein angiography
RVO: Retinal vein occlusion
FAZ: Foveal avascular zone
NPA: Non-perfusion areas
MAs: Microaneurysms
ICC: Intraclass correlation coefficient
BCVA: Best corrected distance visual acuity
IOP: Intraocular pressure
CFP: Color fundus photography
SCP: Superficial capillary plexus
CRVO: Central retinal vein occlusion
BRVO: Branch retinal vein occlusion
NV: Neovascularization
CI: Confidence interval
Log MAR: Logarithm of minimum angle of resolution
